# Weight Gain and Height Growth during Infancy, Childhood, and Adolescence as Predictors of Adult Cardiovascular Risk

**DOI:** 10.1016/j.jpeds.2016.09.059

**Published:** 2017-01

**Authors:** Belavendra Antonisamy, Senthil K. Vasan, Finney S. Geethanjali, Mahasampath Gowri, Y.S. Hepsy, Joseph Richard, P. Raghupathy, Fredrik Karpe, Clive Osmond, Caroline H.D. Fall

**Affiliations:** 1 Department of Biostatistics, Christian Medical College, Vellore, India; 2 Oxford Center for Diabetes, Endocrinology, and Metabolism, University of Oxford, Oxford, United Kingdom; 3 Department of Clinical Biochemistry, Christian Medical College, Vellore, India; 4 Department of Child Health, Christian Medical College, Vellore, India; 5 National Institute for Health Research Oxford Biomedical Research Centre, Oxford University Hospital, Oxford, United Kingdom; 6 Medical Research Council Lifecourse Epidemiology Unit, University of Southampton, Southampton, United Kingdom

**Keywords:** children, weight gain, height gain, conditional growth analysis, cardiovascular risk markers, Asian Indians, BMI, Body mass index, BP, Blood pressure, CVD, Cardiovascular disease, DBP, Diastolic blood pressure, HOMA-IR, Homeostatic model assessment-insulin resistance, LMIC, Low and middle income countries, SBP, Systolic blood pressure, T2DM, Type 2 diabetes mellitus, WC, Waist circumference

## Abstract

**Objectives:**

To investigate independent relationships of childhood linear growth (height gain) and relative weight gain to adult cardiovascular disease (CVD) risk traits in Asian Indians.

**Study design:**

Data from 2218 adults from the Vellore Birth Cohort were examined for associations of cross-sectional height and body mass index (BMI) and longitudinal growth (independent conditional measures of height and weight gain) in infancy, childhood, adolescence, and adulthood with adult waist circumference (WC), blood pressure (BP), insulin resistance (homeostatic model assessment-insulin resistance [HOMA-IR]), and plasma glucose and lipid concentrations.

**Results:**

Higher BMI/greater conditional relative weight gain at all ages was associated with higher adult WC, after 3 months with higher adult BP, HOMA-IR, and lipids, and after 15 years with higher glucose concentrations. Taller adult height was associated with higher WC (men β = 2.32 cm per SD, women β = 1.63, both *P* < .001), BP (men β = 2.10 mm Hg per SD, women β = 1.21, both *P* ≤ .001), and HOMA-IR (men β = 0.08 log units per SD, women β = 0.12, both *P* ≤ .05) but lower glucose concentrations (women β = −0.03 log mmol/L per SD *P* = .003). Greater height or height gain at all earlier ages were associated with higher adult CVD risk traits. These positive associations were attenuated when adjusted for adult BMI and height. Shorter length and lower BMI at birth were associated with higher glucose concentration in women.

**Conclusions:**

Greater height or weight gain relative to height during childhood or adolescence was associated with a more adverse adult CVD risk marker profile, and this was mostly attributable to larger adult size.

Cardiovascular disease (CVD) is the leading cause of death globally, and the population incidence of CVD and related metabolic disorders is higher in low- and middle-income countries (LMICs) than in the rest of the world.^1^, ^2^ Mortality because of premature CVD is increasing in South Asian countries such as India.^1^, ^3^, ^4^

Growth patterns in early life are important predictors of adult CVD risk factors.^4^, ^5^ Lower weight at birth^6^, ^7^ and/or during infancy^8^ and higher weight or BMI during childhood or adolescence^9^, ^10^, ^11^, ^12^ are associated with a higher risk of adult hypertension, type 2 diabetes mellitus (T2DM), and CVD. These relationships may be mediated by effects on body composition; weight and BMI at birth and during infancy positively predict adult lean mass more strongly than adult fat mass, and during late childhood and adolescence, they predict fat mass more strongly.^13^, ^14^, ^15^ Few studies have examined associations of height (linear) growth in early life with later CVD risk.

Understanding when in childhood growth relates to later CVD risk may guide the timing of interventions to prevent disease. Identifying specific ages when linear growth and weight (soft tissue) gain predict later outcomes is complicated by the fact that serial measurements of height or weight within an individual are strongly positively correlated, and height and weight are correlated with each other. Two-way conditional growth measures, which are adjusted for prior size, and mutually adjust weight and height for each other, have been developed to overcome these limitations.^14^ We have used 2-way conditional growth analysis to study independent relationships of linear growth and weight gain, during defined periods of infancy, childhood, and adolescence, with adult CVD risk markers using data from the Vellore birth cohort in India.

## Methods

The Vellore Birth Cohort includes individuals born within defined areas of Vellore town and adjoining rural villages in Tamil Nadu, India during 1969-1973.^9^ The current analysis used data from the 2218 cohort members for whom birth measurements were available and who were followed up as young adults during 1998-2002. Height and weight were measured prospectively by trained research staff, using standardized methods, at birth, during infancy (up to 3 months of age), childhood (6-8 years of age), adolescence (10-15 years of age), and adulthood. Children had up to 3 measurements in the first 3 months of age, up to 2 measurements between 6 and 8 years of age, and up to 5 measurements between 10 and 15 years of age. The study was approved by the institutional ethics committee, and all study participants provided written informed consent.

Adult follow-up took place at a median (IQR) age of 28.1 years (27.4, 28.8).^9^ Measurements included weight to the nearest 0.1 kg; height to the nearest 1 mm, measured using a Harpenden portable stadiometer (Holtain Ltd, Crymych, Dyfed, Wales); and waist circumference (WC) measured to the nearest 1 mm, midway between the costal margin and iliac crest in expiration. Body mass index (BMI) was calculated using the formula weight (kg)/length or height (m) squared. Information was collected on place of residence (both current and at birth), attained education level, current tobacco and alcohol use, physical activity, and socioeconomic status. Education was recorded in 7 groups from no schooling to a professional qualification. Participants were defined as current tobacco users or nonusers. Frequency and quantity of consumption of beer, wine, and spirits were converted into units of alcohol per week. A score was derived as a summary estimate of daily physical activity as described previously.^10^ A 6-point scale ranging from “almost entirely sedentary” to “heavy physical work” was used to classify work-related activity. In addition, the scoring included time spent in domestic and leisure activities and daily mode of transport (walking, cycling). Time periods for each activity were multiplied by metabolic constants derived from published tables of the relative energy expenditure of each task, and summed to create the final physical activity score. Socioeconomic status was assessed by recording possession of up to 15 household items.^11^ Details of anthropometry and CVD risk factors measurements are described elsewhere.^12^

### Data Analyses

#### Analysis Sample

In selecting ages for the growth analysis, we aimed to include (in addition to birth and adulthood) infancy, childhood, and adolescence. The exact ages selected were based upon availability of data. No infant data were collected before March 1, 1971 or in December 1973, and therefore, we excluded births at these times (n = 326), leaving 1892. We also excluded 7 cohort members without birth length and 7 without adult height and weight, leaving 1878.

**Figure 1:**
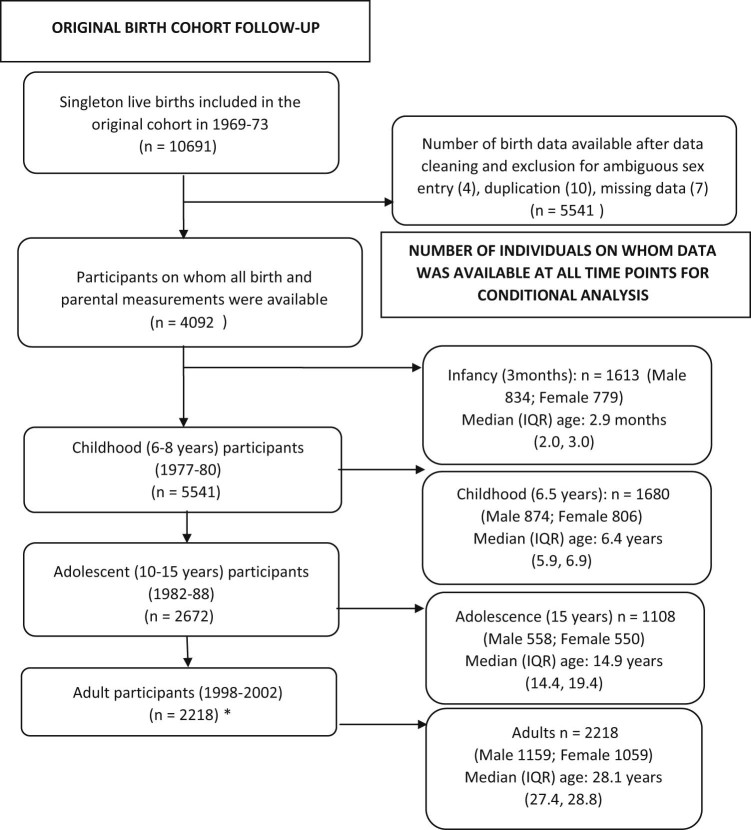
Cohort flow chart. *Number of participants used for cross-sectional analysis.

Among the 1878, infant measurements were available for 1613 (median [IQR] 2.9 months [2.0, 3.0]), childhood measurements for 1680 (6.4 years of age [5.9, 6.9]), and adolescent measurements for 1108 (14.9 years of age [14.4, 19.4]) (Figure 1; available at www.jpeds.com). All measurements were converted into within-cohort age- and sex-specific z scores [(subject mean-cohort mean)/cohort SD]. Exact values at age 3 months, and age 6.5 and 15 years were then obtained by interpolation of the z scores, using the nearest measurements to that age and within 2 months for the infant value and 2 years for the childhood and adolescent values, and back-transformation to the units of measurement.

We examined the associations of weight and height z scores separately, at each age, with each CVD risk marker, using linear regression, first adjusted for adult age alone (model 1) and then by additional adjustment for adult body size (BMI and height, model 2). Model 1 (“forward-looking” approach) addresses the question: What is the net association of size at each age with the adult outcome? Model 2 (“backward-looking” approach) addresses the question: Given that this person has achieved a particular adult BMI and height, is there any remaining effect on the outcome of size at earlier ages, or do the earlier measurements have all their effect through their contribution to adult size? Both models were adjusted for year of birth and sociodemographic variables. We used interaction tests to examine whether associations between body size and cardiovascular risk markers differed between the sexes, and because there were more statistically significant interactions than expected by chance, all analyses were stratified by sex.

We constructed sex-specific and height- and weight-specific conditional variables, which are standardized residuals derived from regressing size z scores at each age on prior size measures.^16^ Conditional height is current height accounting for all prior height and weight measures. Conditional relative weight gain is current weight accounting for current height and all prior weight and height measures. For example, adult conditional relative weight was derived by regressing adult weight on adult height, and weight and height or length at age 15 and 6.5 years, 3 months, and birth.

Conditional relative weight and height gain variables represent children's deviation from expected size based on their own prior measures and on the growth of the other children in the cohort, and can be interpreted as representing greater or less than expected soft tissue gain and linear growth respectively. For example, a child with a positive conditional relative weight at 6.5 years of age is heavier than expected given his/her current height and prior size and, thus, had a faster rate of soft tissue gain from age 3 months to 6.5 years. Again, we created “forward-looking” models adjusted only for adult age (model 1), and “backward-looking” models further adjusted for adult BMI and height (model 2). We included 907 participants who had data at all selected ages for conditional analysis. Analyses were undertaken using SPSS v 22 (SPSS Inc, Chicago, Illinois) and Stata v 13.1 (StataCorp, College Station, Texas).

## Results

When compared with the remainder of the original cohort of 10 691 live singleton births, the 1878 men and women included in this analysis were less likely to have been born to a primiparous mother (studied 11.7%; not studied 19.8%; *P* < .001). There were differences in the education level of the head of the household at the time of their birth, but these were small (illiterate: 12.2% and 12.7%; attended school 5th standard: 36.5% and 32.4%; 6-11 standard: 47.1% and 45.8%; college graduate: 5.4% and 4.1%; *P* < .001). There were no significant differences in parental weight and height. Subjects studied were similar at birth (2795  vs 2815 g; *P* = .19) and heavier at 3 months of age (4858 vs 4706 g; *P* < .001), but there were no significant differences in measurements during childhood. Subjects studied during adolescence were lighter than the subjects not studied though the differences were small.

Early life and adult characteristics of the study sample are shown in Table I. Compared with an international growth reference, they were small at all ages in childhood; mean World Health Organization z scores for length, weight, and BMI at birth were -0.72, -1.17, and -1.28 respectively. Equivalent data for 3 months of age were -1.34, -2.00, and -1.70; for 6.5 years of age -2.27, -2.35, and -1.19 and for 15 years of age -2.11, -2.89, and -1.95. At 28 years of age, the prevalence of underweight (BMI <18.5 kg/m^2^) was 28%, overweight (BMI 25-30 kg/m^2^) 11%, and obesity (BMI >30 kg/m^2^) 2%. Despite a low mean BMI, 14% of men and 17% of women had central obesity defined by the International Diabetes Federation as a waist circumference (WC) >90 cm in men and >80 cm in women.^17^ The prevalence of hypertension, impaired glucose tolerance, and T2DM was high at 9.4%, 16.6%, and 2.8% respectively. Estimates for diastolic blood pressure (DBP) were similar to those for systolic blood pressure (SBP), and are not shown.

### Unadjusted for Adult Size

As expected, length or height at all ages was positively correlated with adult height; correlations strengthened with increasing age of the earlier measurement, from r = 0.28 in men and 0.25 in women for birth length to r = 0.66 in men and 0.79 in women for 15-year height. Similarly BMI at all earlier ages correlated positively with adult BMI (r = 0.13 in men and 0.07 in women for BMI at birth, and 0.52 in both sexes for 15-year BMI).

Taller adult height was associated with higher WC, BP, and homeostatic model assessment-insulin resistance (HOMA-IR) in both sexes, but with lower 120-minute glucose and cholesterol concentrations in women (Table II). Higher adult BMI was associated with higher WC, BP, glucose, cholesterol, and triglyceride concentrations, and HOMA-IR, and lower adult high-density lipoprotein cholesterol concentration in both sexes (Table III). Longer birth length and higher BMI at birth were associated with lower 120-minute glucose concentration among women. Height and BMI at all ages were strongly positively related to adult WC (Table II, Table III). Taller height at 6.5 and 15 years of age was associated with higher BP in both sexes. Taller height at 15 years of age was also associated with higher cholesterol and triglyceride concentrations in men and higher HOMA-IR in women. Higher 15-year BMI was associated with higher BP in both sexes, and with higher cholesterol and triglyceride concentrations in men.

### Adjusted for Adult Size

The positive associations between earlier height and BMI and adult CVD risk markers were attenuated, and most were no longer statistically significant, after adjusting for adult height and BMI (Table II, Table III). Exceptions were BP and cholesterol, which remained positively related to 15-year height. In addition, some inverse associations became apparent. Longer birth length was associated with lower BP, HOMA-IR, and triglyceride concentrations in women (Table II). Higher BMI at birth was associated with lower 120-minute glucose concentration in both sexes (Table III). Higher BMI at 3 months of age was associated with lower WC in women. Higher BMI at 6.5 years of age was associated with lower 120-minute glucose in both sexes, and lower WC, cholesterol, and triglyceride concentrations in men. Taller height at 6.5 years of age was associated with lower triglyceride and higher adult high-density lipoprotein cholesterol concentration in men (Table II). Higher BMI at 15 years of age was associated with lower WC and 120-minute glucose concentration in both sexes, HOMA-IR in men, and cholesterol and triglycerides in women.

### Longitudinal Analysis—Unadjusted for Adult Size

The 907 men and women included in the longitudinal analysis were shorter at birth by 0.48 cm than the 971 not included (*P* < .001) but did not differ significantly in birth weight or height and weight at 3 months, 6.5, 15, or 28 years of age, any of the cardiometabolic risk markers, or rural v urban residence at birth or currently.

**Figure 2:**
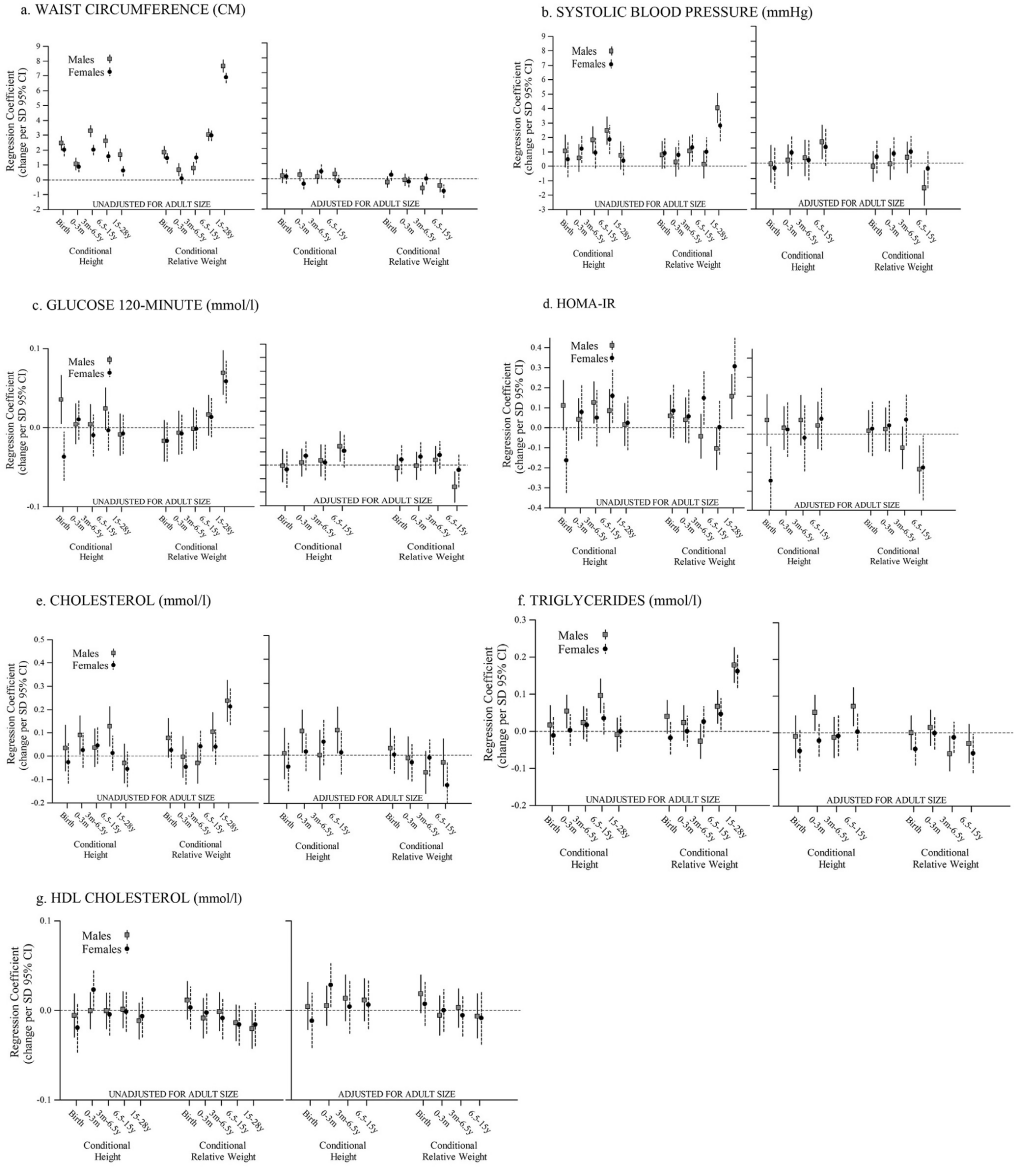
**A**-**G,** Associations of conditional height and conditional relative weight gain from birth to 28 years of age with adult height, BMI, and cardiovascular risk factors, including “forward-looking (unadjusted for adult size)” and “backward-looking (adjusted for adult size)” analyses. Estimates represented as unit increase **B,** in outcome measurement along with 95% CI with unit increase in exposure variable. Gray squares represent men, and black circles represent women.

The results are presented in Figure 2. Greater linear growth at all ages was associated with higher adult WC in both sexes. Greater linear growth between birth and 3 months of age was associated with higher cholesterol and triglyceride concentrations in men, and higher BP in women. Greater linear growth between 3 months and 6.5 years of age was associated with higher BP and HOMA-IR in men, and DBP in women. Greater linear growth from 6.5 to 15 years of age was associated with higher BP in both sexes, higher HOMA-IR in women, and higher total cholesterol and triglycerides in men. Linear growth between 15 and 28 years of age was unrelated to risk factors, and there was little increase in height between these ages (mean 16 cm in men and 5 cm in women) (Table IV; available at www.jpeds.com).

The strongest finding was that greater conditional relative weight gain between 15 and 28 years of age was associated with a more adverse CVD risk profile in both sexes. Greater conditional weight gain at all ages was associated with higher adult WC. Conditional relative weight gain between birth and 3 months of age was unrelated to the risk factors except for WC. Greater conditional relative weight gain between 3 months and 6.5 years of age was associated with higher SBP in both sexes. Greater conditional relative weight gain from 6.5 to 15 years of age was associated with higher triglyceride concentrations in both sexes, higher SBP and DBP in women, and higher total cholesterol in men. The positive associations of conditional relative weight gain between 15 and 28 years of age with risk factors were considerably larger in magnitude than those for conditional relative weight gain before the age of 15 years (Table V; available at www.jpeds.com).

### Adjusted for Adult Size

All the above positive associations were attenuated, and most became nonsignificant after adjusting for adult size. However, greater linear growth between birth and 3 months of age remained associated with higher cholesterol and triglyceride concentrations in men, and higher DBP in women; greater linear growth between 3 months and 6.5 years of age with higher DBP in men; and greater linear growth between 6.5 and 15 years of age with higher BP and total cholesterol and triglyceride concentration in men, and DBP in women.

## Discussion

Overall, our study shows that greater height and BMI after birth and through to adolescence are associated with higher CVD risk factors in adulthood, and these associations are largely attributable to adult height and BMI. The significant differences between males and females were mainly due to differences in the specific ages at which earlier size or growth were related to the outcomes.

Strengths of the study include the use of prospectively recorded growth data and the use of conditional growth analysis, which enabled us to examine independent effects of weight and height growth during specific age periods. Conditional variables are uncorrelated, and expressing them as z scores allows direct comparison of coefficients within regression models. This offers advantages over other representations of growth, and provides more information than weight gain alone. However, we acknowledge that conditional relative weight gain cannot differentiate between lean and fat gain. Very few birth cohorts have longitudinal measurements of lean and fat tissue through childhood, but recent data from a younger South Indian cohort has shown that the adipose component of childhood weight gain contributes most strongly to associations of weight gain with CVD risk markers.^18^ A limitation of the study was cohort attrition, mainly because of deaths and out-migrations; of the original 10 691 live singleton births, we studied 1878 (18%). Those studied differed from the original cohort in a variety of ways (for example infant mortality was higher among lower birth weight individuals). In a within-cohort analysis like this, these differences would not of themselves introduce bias, but would do so only if the associations between early size/growth and cardiometabolic outcomes differed between those studied and not studied. The conditional method requires data at all ages of interest, which led to the further loss of 971 (51.7%) participants from the longitudinal analysis. The remaining 48% of participants did not differ significantly in any early life characteristics, except that they were shorter by 0.48 cm at birth.

Positive associations of BMI and conditional relative weight gain from midchildhood onward with adult risk markers are in agreement with recent studies from LMICs^14^ and developed countries^10^, ^14^ showing that greater BMI gain during childhood and adolescence is associated with an increased risk of adult hypertension, T2DM, metabolic syndrome, and coronary heart disease. Accelerated weight gain, or upward crossing of weight percentiles, at this stage of the life-course is, thus, associated with higher adult risk in all populations studied,^19^ even among children who are relatively light and thin in absolute terms, like the Indian children in our study. Weight gain at this stage of life appears to lead to greater fat than lean gain.^13^, ^20^ Little is being done in any country to measure children in a way that could pick up this harmful growth pattern. If children are monitored at all with future CVD risk in mind, action tends to be limited to the treatment of established obesity, and ignores upward change within the normal BMI range (“children becoming obese relative to themselves”).

Pediatricians in LMICs tend to promote weight gain during infancy because it is known to increase survival and benefit neurodevelopment. This practice has been questioned recently because of data from developed countries showing that rapid infant weight gain is associated with an increased risk of adult obesity and insulin resistance.^21^ However, the Hertfordshire and Finland cohorts showed that greater weight or BMI gain in infancy (under 2 years of age) was associated with a lower risk of coronary heart disease and T2DM^22^, ^23^ suggesting that infancy (like fetal life) is a period of plasticity during which good nutrition improves the development of metabolically active tissues, and build greater lean body mass. Similar concepts have led to the focus on promoting nutrition during “the first 1000 days” (from conception to the end of the second postnatal year) to promote long-term health. We found that weight gain below the age of 3 months was associated with a higher adult WC but not with other adult risk markers. Overall, we conclude that promoting either linear growth or infant weight gain in LMICs is unlikely to increase later CVD risk.

Taller height and faster linear growth during infancy, childhood, and adolescence were associated with higher WC. Positive associations between height or height gain and BP, insulin resistance, cholesterol, and triglycerides were less consistent and mostly explained by greater adult size, although the associations of BP with linear growth 6.5-15 years of age, and of cholesterol with linear growth 0-3 months of age and 6.5-15 years of age in men remained significant after adjustment for adult size. An association of WC with height is not surprising, given that WC is a measure of frame size as well as of central adiposity. An association between BP and height in children is well described and possibly represents a physiological adaptation to perfuse a longer arterial system.^24^ Taller people have a lower risk of CVD, despite higher BP,^25^ so this adaptation may not carry adverse implications for health. Data from the Helsinki cohort has related greater height gain between birth and 7 years of age, to an increased risk of adult hypertension and coronary heart disease.^26^, ^27^, ^28^ Conversely, recent data from the 1946 United Kingdom birth cohort showed that shorter height in early childhood was associated with higher adult carotid intima media thickness.^29^ The association between childhood height growth and insulin resistance may reflect reverse causality; insulin has growth promoting properties, and higher insulin concentrations in childhood may increase height growth. Reasons for the positive association between infant length growth and adult cholesterol and triglyceride concentrations, however, are unclear. Taller childhood height can reflect an accelerated “tempo” of growth, resulting in earlier maturation and puberty. In western populations, earlier puberty has been associated with increased adult CVD risk factors, for reasons that are not clear.^30^, ^31^ Our cohort lacks data on puberty, however, earlier maturation is generally followed by a reduced final height, because of premature epiphyseal fusion, and so this explanation does not fit well with our data, in which WC, BP, and HOMA-IR were positively associated with final height. Future studies examining linear growth in early life in relation to later CVD risk would be useful.

Longer birth length was associated with lower glucose and cholesterol in women, and after adjusting for adult size, with lower HOMA-IR and triglycerides. Higher BMI at birth was associated with lower glucose concentration in women, and in men after adjusting for adult size, similar to previous reports that showed inverse associations between birth weight and T2DM.^7^ Shorter birth length was associated with a higher risk of later T2DM in another Indian birth cohort.^32^ An association between smaller birth size and higher adult BP, found in many birth cohorts,^33^ was not seen in our study, as in other Indian studies.^34^ Associations of small size at birth with later CVD risk markers tend to be weaker in LMICs than in high income settings,^21^ which may reflect less within-cohort heterogeneity in birth weight, because of a lack of newborns in the upper range of birth weight, and/or lower adult BMI in LMICs; in western birth cohorts, the highest prevalence of T2DM is among men and women who were small at birth and became obese adults.

Monitoring childhood weight and height, and active intervention to prevent or reverse upward crossing of BMI percentiles may reduce later CVD risk. Individuals who had greater linear growth during childhood and/or became taller as adults had higher adult WC, BP, insulin resistance, and cholesterol concentrations. It is not clear whether these associations reflect an increased risk of future CVD,^35^, ^36^ and associations between linear growth and adult CVD risk need further investigation. Infant weight gain was positively related to adult WC, but unrelated to BP or the biochemical CVD risk markers, suggesting that the common clinical practice in LMICs of promoting infant weight gain to enhance survival and neurodevelopment is unlikely to have either adverse or beneficial implications for future CVD risk.

## Figures and Tables

**Table IV:** Associations of conditional height from birth to 28 years of age with adult height, BMI, and cardiovascular risk factors, including “forward-looking (model 1)” and “backward-looking (model 2)” analyses

	Men						Women					
	Model 1			Model 2			Model 1			Model 2		
	β*	95% CI	*P* value	β*	95% CI	*P* value	β*	95% CI	*P* value	β*	95% CI	*P* value
Adult height (cm)												
Birth	0.30	0.23,0.37	<.001	-	-	-	0.31	0.25,0.38	<.001	-	-	-
0-3mo	0.26	0.20,0.32	<.001	-	-	-	0.20	0.15,0.25	<.001	-	-	-
3 mo-6.5 y	0.45	0.39,0.50	<.001	-	-	-	0.45	0.39,0.50	<.001	-	-	-
6.5-15 y	0.34	0.28,0.40	<.001	-	-	-	0.40	0.35,0.45	<.001	-	-	-
15-28 y												
BMI(kg/m^2^)												
Birth	0.10	0.02,0.19	<.001	-	-	-	0.15	0.07,0.24	.001	-	-	-
0-3 mo	0.00	-0.07,0.08	.9	-	-	-	0.06	-0.01,0.13	.1	-	-	-
3 mo-6.5 y	0.20	0.13,0.28	<.001	-	-	-	0.07	-0.005,0.14	.06	-	-	-
6.5-15 y	0.12	0.04,0.19	.003	-	-	-	0.10	0.03,0.17	.005	-	-	-
15-28 y												
WC(cm)												
Birth	2.47	2.00,2.93	<.001	0.19	-0.26,0.64	.4	2.01	1.59,2.42	<.001	0.14	-0.31,0.59	.5
0-3 mo	1.06	0.67,1.45	<.001	0.24	-0.14,0.61	.2	0.87	0.53,1.21	<.001	-0.34	-0.70,0.01	.06
3 mo-6.5 y	3.28	2.89,3.66	<.001	0.12	-0.31,0.55	.5	2.03	1.67,2.39	<.001	0.48	0.03,0.92	.04
6.5-15 y	2.60	2.20,3.00	<.001	0.30	-0.11,0.70	.1	1.56	1.22,1.90	<.001	-0.19	-0.60,0.23	.3
15-28 y	1.67	1.28,2.07	<.001	-	-	-	0.6	0.26,0.95	<.001	-	-	-
SBP(mm Hg)												
Birth	1.04	-0.08,2.15	.07	-0.05	-1.26,1.17	.9	0.45	-0.74,1.64	.4	-0.34	-1.67,0.98	.6
0-3 mo	0.57	-0.37,1.50	.2	0.19	-0.83,1.21	.7	1.21	0.23,2.19	.02	0.69	-0.36,1.73	.2
3 mo-6.5 y	1.83	0.90,2.75	<.001	0.34	-0.84,1.52	.5	0.91	-0.12,1.94	.08	0.19	-1.11,1.49	.7
6.5-15 y	2.46	1.50,3.42	<.001	1.37	0.27,2.48	.02	1.84	0.87,2.82	<.001	1.06	-0.15,2.27	.09
15-28 y	0.73	-0.21,1.68	.1	-	-	-	0.38	-0.61,1.37	.4	-	-	-
DBP(mm Hg)												
Birth	1.42	0.52,2.31	<.001	1.00	0.03,1.97	.04	0.23	-0.68,1.15	.6	-0.36	-1.38,0.66	.4
0-3 mo	0.41	-0.34,1.16	.2	0.49	-0.31,1.31	.2	1.34	0.64,2.15	.002	1.01	0.20,1.82	.01
3 mo-6.5 y	1.41	0.75,2.39	<.001	0.95	0.02,1.89	.04	0.87	0.07,1.66	.03	0.51	-0.48,1.52	.3
6.5-15 y	1.95	1.18,2.72	<.001	1.59	0.71,2.47	<.001	1.65	0.90,2.39	<.001	1.19	0.25,2.12	.01
15-28 y	-0.31	-1.06,0.45	.4	-	-	-	-0.15	-0.91,0.60	.6	-	-	-
Log glucose 120 min(mmol/L)												
Birth	0.04	0.01,0.07	.02	0.03	-0.01,0.06	.1	-0.04	-0.07,-0.01	.02	-0.05	-0.08,-0.01	.01
0-3 mo	0.004	-0.02,0.03	.7	0.01	-0.02,0.04	.5	0.01	-0.02,0.03	.4	0.004	-0.02,0.03	.7
3 mo-6.5 y	0.004	-0.02,0.03	.7	-0.01	-0.04,0.02	.6	-0.01	-0.04,0.02	.4	-0.01	-0.05,0.02	.4
6.5-15 y	0.02	-0.003,0.05	.08	0.02	-0.01,0.05	.2	-0.004	-0.03,0.02	.7	-0.01	-0.04,0.02	.5
15-28 y	-0.01	-0.04,0.02	.4	-	-	-	-0.01	-0.03,0.02	.5	-	-	-
HOMA-IR												
Birth	0.11	-0.01,0.24	.08	0.07	-0.06,0.21	.2	-0.16	-0.32,0.001	.05	-0.25	-0.43,-0.07	.01
0-3 mo	0.04	-0.06,0.15	.4	0.03	-0.08,0.15	.5	0.08	-0.06,0.21	.2	0.02	-0.12,0.17	.7
3 mo-6.5 y	0.13	0.02,0.23	.02	0.07	-0.06,0.21	.2	0.05	-0.09,0.19	.4	-0.02	-0.19,0.16	.8
6.5-15 y	0.08	-0.02,0.19	.1	0.05	-0.08,0.17	.4	0.16	0.03,0.29	.02	0.08	-0.08,0.24	.3
15-28 y	0.02	-0.09,0.12	.7	-	-	-	0.02	-0.11,0.16	.7	-	-	-
Cholesterol(mmol/L)												
Birth	0.04	-0.06,0.13	.4	0.01	-0.10,0.11	.8	-0.03	-0.12,0.06	.5	-0.05	-0.15,0.05	.3
0-3 mo	0.09	0.01,0.17	.03	0.1	0.01,0.19	.03	0.03	-0.05,0.10	.4	0.01	-0.06,0.09	.7
3 mo-6.5 y	0.04	-0.05,0.12	.3	0.00	-0.10,0.10	.9	0.05	-0.03,0.12	.2	0.06	-0.04,0.15	.2
6.5-15 y	0.13	0.04,0.21	<.001	0.11	0.01,0.20	.03	0.01	-0.06,0.09	.7	0.01	-0.08,0.15	.8
15-28 y	-0.03	-0.11,0.05	.4	-	-	-	-0.06	-0.13,0.02	.1	-	-	-
Log triglycerides(mmol/L)												
Birth	0.02	-0.04,0.07	.5	-0.01	-0.07,0.05	.7	-0.01	-0.06,0.04	.6	-0.05	-0.11,0.01	.08
0-3 mo	0.05	0.01,0.10	.02	0.05	0.01,0.10	.03	0.002	-0.04,0.04	.9	-0.02	-0.07,0.02	.3
3 mo-6.5 y	0.02	-0.02,0.07	.3	-0.01	-0.07,0.04	.6	0.02	-0.03,0.06	.4	-0.009	-0.06,0.05	.7
6.5-15 y	0.10	0.05,0.14	<.001	0.07	0.02,0.12	.01	0.03	-0.01,0.07	.1	0.002	-0.05,0.05	.9
15-28 y	-0.01	-0.05,0.03	.6	-	-	-	0.00	-0.04,0.04	.9	-	-	-
HDL cholesterol(mmol/L)												
Birth	-0.01	-0.03,0.02	.6	0.004	-0.02,0.03	.7	-0.02	-0.05,0.01	.1	-0.01	-0.04,0.02	.4
0-3 mo	-0.001	-0.02,0.02	.9	0.005	-0.02,0.03	.6	0.02	0.000,0.05	.05	0.03	0.00,0.05	.02
3 mo-6.5 y	-0.001	-0.02,0.02	.9	0.01	-0.01,0.04	.30	-0.005	-0.03,0.02	.7	0.004	-0.03,0.03	.8
6.5-15 y	0.001	-0.02,0.02	.1	0.01	-0.01,0.04	.3	-0.002	-0.02,0.02	.8	0.01	-0.02,0.03	.6
15-28 y	-0.01	-0.03,0.01	.2	-	-	-	-0.01	-0.03,0.02	.5	-	-	-

β* represents unit change in outcome variable with unit change in exposure.

For each risk factor,all 10 conditional variables were included together in regression models. Model 1 was adjusted for adult age; model 2 was additionally adjusted for adult size(BMI and height). All models were adjusted for sociodemographic variables(rural or urban residence at birth,rural or urban residence currently,attained education level,adult household possessions score,smoking history,physical activity,and alcohol consumption).

**Table V:** Associations of conditional relative weight gain from birth to 28 years of age with adult height, BMI, and cardiovascular risk factors, including “forward-looking (model 1)” and “backward-looking (model 2)” analyses

	Men						Women					
	Model 1			Model 2			Model 1			Model 2		
	β*	95% CI	*P* value	β*	95% CI	*P* value	β*	95% CI	*P* value	β*	95% CI	*P* value
Adult height (cm)												
Birth	0.09	0.03,0.15	.003	-	-	-	0.11	0.06,0.17	<.001	-	-	-
z weight 0-3 mo	0.10	0.04,0.16	.001	-	-	-	0.16	0.11,0.21	<.001	-	-	-
z weight 3 mo-6.5 y	0.00	-0.06,0.06	.1	-	-	-	-0.02	-0.08,0.03	.4	-	-	-
z weight 6.5-15 y	-0.24	-0.29,-0.18	<.001	-	-	-	-0.28	-0.33,-0.23	<.001	-	-	-
BMI(kg/m^2^)												
Birth	0.18	0.11,0.26	<.001	-	-	-	0.12	0.04,0.19	.002	-	-	-
0-3mo	0.07	-0.004,0.15	.07	-	-	-	0.04	-0.03,0.11	.3	-	-	-
3 mo-6.5 y	0.14	0.06,0.22	<.001	-	-	-	0.20	0.13,0.27	<.001	-	-	-
6.5-15 y	0.40	0.33,0.48	<.001	-	-	-	0.46	0.39,0.54	<.001	-	-	-
WC(cm)												
Birth	1.85	1.46,2.25	<.001	-0.23	-0.59,0.14	.2	1.46	1.11,1.82	<.001	0.24	-0.12,0.59	.1
0-3 mo	0.67	0.26,1.09	<.001	-0.09	-0.46,0.29	.6	0.07	-0.27,0.41	.6	-0.2	-0.55,0.15	.2
3 mo-6.5 y	0.78	0.37,1.19	<.001	-0.65	-1.02,-0.28	<.001	1.49	1.15,1.83	<.001	-0.02	-0.36,0.32	.9
6.5-15 y	3.04	2.65 3.43	<.001	-0.46	-0.87,-0.04	.03	2.97	2.63,3.32	<.001	-0.82	-1.24,-0.40	<.001
15-28 y	7.67	7.25,8.08	<.001	-	-	-	6.89	6.53,7.25	<.001	-	-	-
SBP(mm Hg)												
Birth	0.78	-0.16,1.73	.1	-0.22	-1.21,0.77	.6	0.89	-0.12,1.90	.08	0.39	-0.65,1.43	.4
0-3 mo	0.28	-0.72,1.28	.5	-0.06	-1.08,0.96	.9	0.78	-0.20,1.76	.1	0.63	-0.39,1.65	.2
3 mo-6.5 y	1.06	0.07,2.04	.04	0.37	-0.64,1.37	.4	1.30	0.33,2.27	.01	0.74	-0.26,1.74	.1
6.5-15 y	0.11	-0.83,1.05	.8	-1.63	-2.77,-0.50	<.001	0.98	-0.01,1.97	.05	-0.38	-1.63,0.87	.5
15-28 y	4.03	3.03,5.03	<.001	-	-	-	2.79	1.75,3.83	<.001	-	-	-
DBP(mm Hg)												
Birth	0.60	-0.16,1.36	.1	-0.07	-0.86,0.71	.8	0.50	-0.27,1.28	.2	0.05	-0.74,0.86	.8
0-3 mo	0.61	-0.18,1.41	.1	0.47	-0.33,1.29	.2	0.44	-0.30,1.20	.2	0.47	-0.31,1.25	.2
3 mo-6.5 y	0.24	-0.55,1.03	.5	-0.30	-1.10,0.49	.4	0.66	-0.07,1.41	.07	0.003	-0.76,0.77	.9
6.5-15 y	0.26	-0.49,1.02	.4	-1.49	-2.39,-0.59	.001	1.09	0.33,1.85	.005	-0.76	-1.72,0.20	.1
15-28 y	3.08	2.27,3.88	<.001	-	-	-	3.09	2.29,3.89	<.001	-	-	-
Log glucose 120 min(mmol/L)												
Birth	-0.02	-0.04,0.01	.2	-0.03	-0.06,-0.01	.02	-0.02	-0.04,0.01	.1	-0.03	-0.05,0.00	.06
0-3 mo	-0.01	-0.03,0.02	.6	-0.01	-0.04,0.02	.4	-0.01	-0.03,0.02	.5	-0.01	-0.03,0.02	.6
3 mo-6.5 y	0.00	-0.03,0.03	.8	-0.01	-0.04,0.01	.2	0.00	-0.03,0.02	.8	-0.02	-0.04,0.01	.2
6.5-15 y	0.02	-0.01,0.04	.2	-0.03	-0.06,0.00	.09	0.01	-0.01,0.04	.3	-0.03	-0.06,0.00	.09
15-28 y	0.07	0.04,0.10	<.001	-	-	-	0.06	0.03,0.08	<.001	-	-	-
HOMA-IR												
Birth	0.06	-0.05,0.16	.2	0.02	-0.09,0.13	.7	0.08	-0.05,0.22	.2	0.03	-0.11,0.17	.6
0-3 mo	0.04	-0.07,0.15	.4	0.03	-0.09,0.14	.6	0.06	-0.08,0.19	.4	0.05	-0.09,0.18	.5
3 mo-6.5 y	-0.04	-0.15,0.07	.4	-0.07	-0.18,0.04	.2	0.15	0.02,0.28	.07	0.08	-0.06,0.21	.2
6.5-15 y	-0.11	-0.21,0.000	.05	-0.19	-0.31,-0.06	<.001	0.00	-0.13,0.14	.9	-0.18	-0.35,-0.01	.04
15-28 y	0.16	0.05,0.27	.01	-	-	-	0.31	0.17,0.45	<.001	-	-	-
Cholesterol(mmol/L)												
Birth	0.08	-0.01,0.16	.0	0.03	-0.06,0.11	.5	0.03	-0.05,0.10	.5	0.00	-0.08,0.08	.9
0-3 mo	0.00	-0.09,0.08	.9	-0.01	-0.10,0.08	.8	-0.05	-0.12,0.03	.2	-0.03	-0.11,0.05	.4
3 mo-6.5 y	-0.03	-0.12,0.06	.4	-0.07	-0.16,0.02	.1	0.04	-0.03,0.11	.2	-0.01	-0.09,0.06	.7
6.5-15 y	0.10	0.02,0.19	.01	-0.03	-0.13,0.07	.5	0.04	-0.04,0.11	.3	-0.12	-0.22,-0.03	.01
15-28 y	0.24	0.15,0.33	<.001	-	-	-	0.21	0.13,0.29	<.001	-	-	-
Log triglycerides (mmol/L)												
Birth	0.04	-0.01,0.08	.0	0.001	-0.05,0.05	.9	-0.02	-0.06,0.02	.3	-0.05	-0.09,-0.002	.04
0-3 mo	0.02	-0.03,0.07	.3	0.01	-0.03,0.06	.5	0.000	-0.04,0.04	.9	-0.002	-0.05,0.04	.9
3 mo-6.5 y	-0.03	-0.07,0.02	.2	-0.06	-0.10,-0.01	.02	0.03	-0.02,0.07	.2	-0.01	-0.05,0.03	.5
6.5-15 y	0.07	0.02,0.11	<.001	-0.03	-0.08,0.02	.2	0.05	0.004,0.09	.03	-0.06	-0.11,-0.01	.03
15-28 y	0.18	0.13,0.23	<.001	-	-	-	0.16	0.12,0.20	<.001	-	-	-
HDL cholesterol (mmol/L)												
Birth	0.01	-0.01,0.03	.3	0.02	-0.003,0.04	.1	0.003	-0.02,0.03	.8	0.01	-0.02,0.03	.5
0-3 mo	-0.01	-0.03,0.01	.4	-0.01	-0.03,0.02	.6	-0.003	-0.03,0.02	.8	0.000	-0.02,0.02	.9
3 mo-6.5 y	-0.002	-0.02,0.02	.8	0.003	-0.02,0.02	.8	-0.01	-0.03,0.01	.4	-0.01	-0.03,0.02	.6
6.5-15 y	-0.01	-0.03,0.01	.1	-0.01	-0.03,0.02	.5	-0.02	-0.04,0.01	.1	-0.01	-0.04,0.02	.5
15-28 y	-0.02	-0.04,0.000	.05	-	-	-	-0.02	-0.04,0.01	.1	-	-	-

β* represents unit change in outcome variable with unit change in exposure.

For each risk factor,all 10 conditional variables were included together in regression models. Model 1 was adjusted for adult age; model 2 was additionally adjusted for adult size(BMI and height). All models were adjusted for sociodemographic variables(rural or urban residence at birth,rural or urban residence currently,attained education level,adult household possessions score,smoking history,physical activity,and alcohol consumption).

**Table I:** Characteristics of the study participants

	n	Males	n	Females
Height (cm)				
Birth	981	48.4(2.7)	897	47.9(2.8)
3 mo	834	58.4(3.1)	779	57.4(3.0)
6.5 y	874	107.0(5.4)	806	106.3(5.5)
15 y	558	150.5(9.0)	550	148.8(6.1)
Adult	981	166.4(6.6)	897	153.9(5.9)
Weight(kg)				
Birth	981	2.84(0.49)	897	2.75(0.49)
3 mo	834	4.99(0.82)	779	4.68(0.72)
6.5 y	874	16.0(2.0)	806	15.8(2.0)
15 y	558	35.7(6.7)	550	37.3(6.0)
Adult	981	57.2(11.2)	897	49.1(10.4)
BMI(kg/m^2^)				
Birth	981	12.1(1.8)	897	12.0(1.8)
3 mo	834	14.7(2.1)	779	14.2(1.9)
6.5 y	874	13.9(1.4)	806	14.0(1.4)
15 y	558	15.6(1.5)	550	16.8(2.0)
Adult	981	20.6(3.4)	897	20.7(4.0)
Adult variables				
Age (y)	981	28.0(1.0)	897	28.2(0.9)
WC (cm)	981	77.3(10.5)	897	70.8(9.4)
Hip circumference(cm)	981	86.8(7.3)	897	88.7(8.7)
SBP (mm Hg)	981	112.2(11.2)	897	101.4(10.8)
DBP (mm Hg)	981	72.7(9.3)	897	72.4(8.6)
Total cholesterol (mmol/L)	981	4.0(0.9)	897	3.9(0.8)
HDL cholesterol (mmol/L)	970	0.98(0.24)	893	1.09(0.24)
LDL cholesterol (mmol/L)	968	2.5(0.7)	893	2.4(0.7)
Triglyceride (mmol/L)*	980	1.0(0.8,1.5)	897	0.8(0.6,1.1)
Fasting glucose (mg/dL)*	981	98(92,104)	897	96(90,102)
2-h glucose(mg/dL)*	981	110(94,131)	897	115(99,134)
Fasting insulin (mIU/mL)*	981	5.7(2.6,9.7)	897	3.2(1.2,6.2)
2-h insulin (mIU/mL)*	981	21.7(11.9,39.1)	897	20.8(10.7,37.3)
HOMA-IR*	981	1.35(0.60,2.41)	897	0.77(0.28,1.49)
Urban at birth(%)	981	29.7	897	26.1
Urban as adult(%)	981	45.7	897	41.8
Education: ≤ primary (%)	981	18	897	36.7
Education: middle and high (%)	981	57.1	897	48.9
Education: > high (%)	981	24.9	897	14.3
Material possessions score	981	5(3.8)	897	5(2.7)
Current smoker (%)	981	42.9	897	2.7
Alcohol consumption: any (%)	981	54	897	0.8
Physical activity score	981	1407(832,1890)	897	1854(1427,2409)

*HDL*,high-density lipoprotein; *LDL*,low-density lipoprotein.

“High” education refers to higher secondary school,diplomas,other graduates,and higher degrees.

* For skewed variables,data are presented as median and IQR.

**Table II:** Cross-sectional analysis of z score height from birth to adulthood and adult cardiovascular risk outcome**s**

	Men				Women			
	Model 1		Model 2		Model 1		Model 2	
	β (95% CI)	*P* value	β (95% CI)	*P* value	β (95% CI)	*P* value	β (95% CI)	*P* value
WC(cm)								
Birth	1.27(0.60,1.94)	<.001	-0.13(-0.40,0.15)	.4	0.99(0.37,1.62)	.002	-0.14(-0.42,0.14)	.3
3 mo	0.99(0.32,1.66)	.004	0.04(-0.24,0.31)	.8	0.90(0.30,1.49)	.003	-0.43(-0.69,-0.16)	.002
6.5 y	2.57(1.94,3.21)	<.001	0.01(-0.31,0.34)	.9	1.69(1.09,2.29)	<.001	0.14(-0.16,0.45)	.4
15 y	3.51(2.71,4.31)	<.001	0.35(-0.09,0.80)	.1	3.10(2.33,3.88)	<.001	-0.21(-0.74,0.32)	.4
28 y	2.32(1.70,2.94)	<.001	-		1.63(1.04,2.21)	<.001	-	
SBP (mm Hg)								
Birth	0.55(-0.21,1.30)	.2	-0.36(-1.10,0.37)	.3	-0.37(-1.15,-0.41)	.4	-1.03(-1.80,-0.27)	.008
3 mo	0.52(-0.22,1.26)	.2	-0.27(-1.00,0.46)	.5	0.59(-0.16,1.33)	.1	-0.11(-0.86,0.65)	.8
6.5 y	1.65(0.90,2.40)	<.001	-0.16(-1.03,0.70)	.7	1.10(0.33,1.89)	.006	0.10(-0.76,0.97)	.8
15 y	2.89(1.95,3.82)	<.001	1.53(0.29,2.76)	.02	2.55(1.54,3.57)	<.001	1.28(-0.20,2.76)	.09
28 y	2.10(1.40,2.80)	<.001	-		1.21(0.48,1.95)	.001	-	
Log glucose 120-min(mmol/L)								
Birth	0.01(-0.01,0.03)	.3	0.01(-0.01,0.03)	.5	-0.02(-0.04,-0.00)	.04	-0.2(-0.04,0.00)	.06
3 mo	0.01(-0.01,0.03)	.3	0.01(-0.01,0.03)	.2	0.01(-0.01,0.02)	.6	0.01(-0.01,0.03)	.3
6.5 y	-0.00(-0.02,0.02)	.8	-0.01(-0.04,0.01)	.3	-0.02(-0.03,0.00)	.1	-0.01(-0.03,0.01)	.4
15 y	0.02(-0.01,0.04)	.2	0.01(-0.03,0.04)	.7	-0.02(-0.05,0.00)	.08	-0.02(-0.06,0.02)	.3
28 y	-0.01(-0.03,0.01)	.3	-		-0.03(-0.04,-0.01)	.003	-	
Log HOMA-IR								
Birth	0.07(-0.01,0.15)	.1	0.04(-0.05,0.12)	.4	-0.05(-0.15,0.05)	.4	-0.11(-0.21,0.01)	.03
3 mo	-0.05(-0.15,0.05)	.4	0.03(-0.06,0.12)	.5	0.08(-0.02,0.17)	.1	0.01(-0.09,0.11)	.8
6.5 y	0.08(-0.02,0.17)	.1	-0.02(-0.12,0.08)	.7	0.07(-0.04,0.17)	.2	-0.03(-0.15,0.08)	.6
15 y	0.07(-0.04,0.17)	.2	-0.02(-0.15,0.12)	.8	0.16(0.02,0.29)	.03	-0.02(-0.22,0.18)	.8
28 y	0.08(0.00,0.16)	.05	-		0.12(0.02,0.21)	.02	-	
Cholesterol(mmol/L)								
Birth	-0.01(-0.07,0.05)	.7	-0.04(-0.10,0.02)	.2	-0.02(-0.07,-0.04)	.6	-0.02(-0.08,0.04)	.5
3 mo	0.03(-0.03,0.09)	.3	0.03(-0.03,0.09)	.4	0.01(-0.05,0.06)	.8	-0.00(-0.06,0.06)	1.0
6.5 y	0.05(-0.01,0.11)	.1	-0.01(-0.08,0.06)	.7	-0.11(-0.06,0.06)	.9	0.01(-0.06,0.07)	.8
15 y	0.13(0.05,0.20)	.002	0.11(0.00,0.21)	.04	-0.01(-0.09,0.07)	.8	-0.02(-0.13,0.09)	.7
28 y	0.00(-0.05,0.06)	.9	-		-0.06(-0.11,-0.00)	.05	-	
Log triglycerides(mmol/L)								
Birth	-0.01(-0.04,0.03)	.8	-0.03(-0.06,0.01)	.1	-0.02(-0.06,0.01)	.2	-0.04(-0.07,-0.01)	.02
3 mo	0.02(-0.02,0.05)	.3	0.01(-0.02,0.05)	.6	0.00(-0.03,0.03)	.9	-0.02(-0.05,0.02)	.3
6.5 y	0.01(-0.04,0.03)	.8	-0.07(-0.11,-0.03)	.001	0.00(-0.03,0.03)	1.0	-0.01(-0.05,0.02)	.5
15 y	0.06(0.01,0.10)	.01	0.03(-0.03,0.09)	.3	0.03(-0.02,0.07)	.2	-0.01(-0.08,0.05)	.7
28 y	0.02(-0.02,0.05)	.3	-		-0.01(-0.04,0.02)	.6	-	
HDL cholesterol(mmol/L)								
Birth	-0.00(-0.02,0.01)	.8	0.00(-0.01,0.02)	.7	-0.02(-0.03,0.00)	.1	-0.01(-0.03,0.01)	.3
3 mo	0.01(-0.01,0.02)	.6	0.01(-0.01,0.03)	.3	0.00(-0.02,0.02)	.8	0.01(-0.01,0.03)	.3
6.5 y	0.01(-0.01,0.03)	.2	0.03(0.01,0.05)	.009	-0.00(-0.02,0.01)	.7	0.01(-0.01,0.03)	.5
15 y	0.01(-0.01,0.03)	.3	0.03(-0.00,0.05)	.06	-0.01(-0.03,0.02)	.5	0.02(-0.02,0.05)	.3
28 y	-0.01(-0.03,0.1)	.2	-		-0.01(-0.03,0.00)	.1	-	

Separate regression analyses were performed for height at each age. β represents unit change in outcome variable with unit change in z height. Number of individuals at each time point were: (males): 981 at birth, 834 at 3 months, 874 at 6.5 years, 558 at 15 years, and 981 at 28 years; and (females): 897 at birth, 779 at 3 months, 806 at 6.5 years, 550 at 15 years, and 897 at 28 years. Model 1 was adjusted for adult age, Model 2 was additionally adjusted for adult size (BMI and height). Other covariates included in both models were age, rural/urban residence (at birth and currently), education, household material possessions, smoking, alcohol consumption, and physical activity.

**Table III:** Cross-sectional analysis of z score BMI from birth to adulthood and adult cardiovascular risk outcomes

	Men				Women			
	Model 1		Model 2		Model 1		Model 2	
	β (95% CI)	*P* value	β (95% CI)	*P* value	β (95% CI)	*P* value	β (95% CI)	*P* value
WC(cm)								
Birth	0.95(0.27,1.63)	.007	-0.21(-0.48,0.05)	.1	0.67(0.06,1.29)	.03	0.04(-0.22,0.31)	.8
3 mo	1.22(0.53,1.91)	.001	-0.18(-0.45,0.09)	.2	0.89(0.23,1.55)	.009	-0.08(-0.37,0.20)	.6
6.5 y	0.66(0.03,1.29)	.04	-0.46(-0.72,-0.20)	<.001	0.99(0.39,1.60)	.001	-0.01(-0.27,0.26)	1.0
15 y	4.14(3.66,5.17)	<.001	-0.57(-0.94,-0.20)	.003	3.49(2.82,4.16)	<.001	-0.70(-1.07,-0.33)	<.001
28 y	9.10(8.79,9.41)	<.001	-		8.22(7.91,8.53)	<.001	-	
SBP(mm Hg)								
Birth	-0.02(-0.78,0.75)	1.0	-0.54(-1.26,0.18)	.1	0.42(-0.35,1.19)	.3	0.14(-0.59,0.87)	.7
3 mo	0.27(-0.50,1.03)	.5	-0.47(-1.19,0.26)	.2	0.68(-0.14,1.51)	.1	0.23(-0.57,1.03)	.6
6.5 y	0.30(-0.43,1.02)	.4	-0.04(-0.73,0.65)	.9	0.20(-0.58,0.99)	.6	-0.15(-0.91,0.61)	.7
15 y	1.60(0.65,2.54)	.001	-0.62(-1.66,0.43)	.2	1.74(0.81,2.66)	<.001	0.17(-0.89,1.22)	.8
28 y	3.91(3.22,4.60)	<.001	-		3.59(2.84,4.33)	<.001	-	
Log glucose 120-min(mmol/L)								
Birth	-0.01(-0.03,0.01)	.2	-0.02(-0.04,-0.00)	.03	-0.02(-0.04,0.00)	.05	-0.02(-0.04,-0.00)	.02
3 mo	-0.00(-0.02,0.02)	.9	-0.01(-0.03,0.01)	.4	-0.01(-0.03,0.01)	.3	-0.01(-0.03,0.01)	.4
6.5 y	-0.01(-0.03,0.01)	.3	-0.02(-0.04,0.01)	.01	-0.02(-0.03,0.00)	.1	-0.03(-0.04,-0.01)	.007
15 y	0.00(-0.02,0.03)	.9	-0.04(-0.07,-0.01)	.003	0.00(-0.02,0.02)	1	-0.03(-0.06,-0.01)	.01
28 y	0.07(0.05,0.09)	<.001	-		0.05(0.03,0.06)	<.001	-	
HOMA-IR								
Birth	0.06(-0.03,0.14)	.2	0.03(-0.05,0.12)	.4	0.05(-0.04,0.15)	.3	0.03(-0.07,0.13)	.6
3 mo	0.03(-0.05,0.12)	.5	-0.00(-0.09,0.08)	1.0	-0.01(-0.12,0.10)	.8	-0.06(-0.16,0.05)	.3
6.5 y	-0.03(-0.11,0.05)	.5	-0.05(-0.12,0.03)	.3	0.08(-0.02,0.18)	.1	0.05(-0.05,0.15)	.4
15 y	-0.03(-0.13,0.07)	.6	-0.12(-0.23,-0.01)	.04	0.09(-0.03,0.21)	.1	-0.11(-0.25,0.03)	.1
28 y	0.18(0.10,0.26)	<.001	-		0.29(0.19,0.38)	<.001	-	
Cholesterol(mmol/L)								
Birth	-0.00(-0.06,0.06)	1.0	-0.03(-0.09,0.03)	.3	0.02(-0.04,0.08)	.5	0.01(-0.05,0.06)	.8
3 mo	-0.00(-0.07,0.06)	.9	-0.04(-0.10,0.02)	.2	-0.00(-0.06,0.06)	1.0	-0.02(-0.08,0.04)	.6
6.5 y	-0.01(-0.07,0.04)	.6	-0.06(-0.11,0.00)	.05	0.01(-0.05,0.06)	.9	-0.03(-0.09,0.02)	.3
15 y	0.13(0.06,0.21)	.001	-0.02(-0.10,0.07)	.7	0.03(-0.04,0.10)	.4	-0.10(-0.18,-0.02)	.01
28 y	0.28(0.23,0.34)	<.001	-		0.21(0.15,0.27)	<.001	-	
Log triglycerides(mmol/L)								
Birth	-0.00(-0.04,0.03)	.9	-0.03(-0.06,0.01)	.1	-0.00(-0.03,0.03)	.8	-0.02(-0.05,0.02)	.3
3 mo	0.02(-0.02,0.05)	.4	-0.01(-0.05,0.02)	.5	0.00(-0.03,0.04)	1.0	-0.02(-0.05,0.02)	.4
6.5 y	-0.01(-0.05,0.02)	.5	-0.04(-0.07,-0.01)	.02	0.02(-0.01,0.05)	.3	-0.01(-0.04,0.02)	.6
15 y	0.07(0.03,0.12)	.001	-0.04(-0.09,0.01)	.1	0.03(-0.01,0.07)	.1	-0.08(-0.12,-0.03)	.001
28 y	0.20(0.16,0.23)	<.001	-		0.17(0.13,0.20)	<.001	-	
HDL cholesterol(mmol/L)								
Birth	0.00(-0.02,0.02)	.8	0.01(-0.01,0.02)	.5	0.00(-0.01,0.02)	.7	0.01(-0.01,0.02)	.4
3 mo	-0.00(-0.02,0.02)	.8	0.00(-0.02,0.02)	.8	-0.01(-0.03,0.01)	.3	-0.01(-0.02,0.01)	.6
6.5 y	0.00(-0.01,0.02)	.6	0.01(-0.01,0.02)	.3	-0.01(-0.03,0.01)	.2	-0.01(-0.03,0.01)	.3
15 y	-0.01(0.03,0.01)	.5	0.01(-0.01,0.04)	.3	-0.02(-0.04,0.01)	.1	0.00(-0.02,0.03)	.8
28 y	-0.02(-0.04,-0.01)	<.001	-		-0.04(-0.05,-0.02)	<.001	-	

Separate regression analyses were performed for BMI at each age. β represents unit change in outcome variable with unit change in z BMI. Total number of individuals at each time point were: (males) 981 at birth, 834 at 3 months, 874 at 6.5 years, 558 at 15 years, and 981 at 28 years; and (females): 897 at birth, 779 at 3 months, 806 at 6.5 years, 550 at 15 years, and 897 at 28 years. Model 1 was adjusted for adult age, Model 2 was additionally adjusted for adult size (BMI and height). Other covariates included in both models were age, rural/urban residence (at birth and currently), education, household material possessions, smoking, alcohol consumption, and physical activity.
